# Supplementary figures and images for: mTORC1 is required for differentiation of germline stem cells in the Drosophila melanogaster testis
Source: PLoS One. 2024 Mar 21;19(3):e0300337. doi: 10.1371/journal.pone.0300337 (PMC10956854; doi:10.1371/journal.pone.0300337)

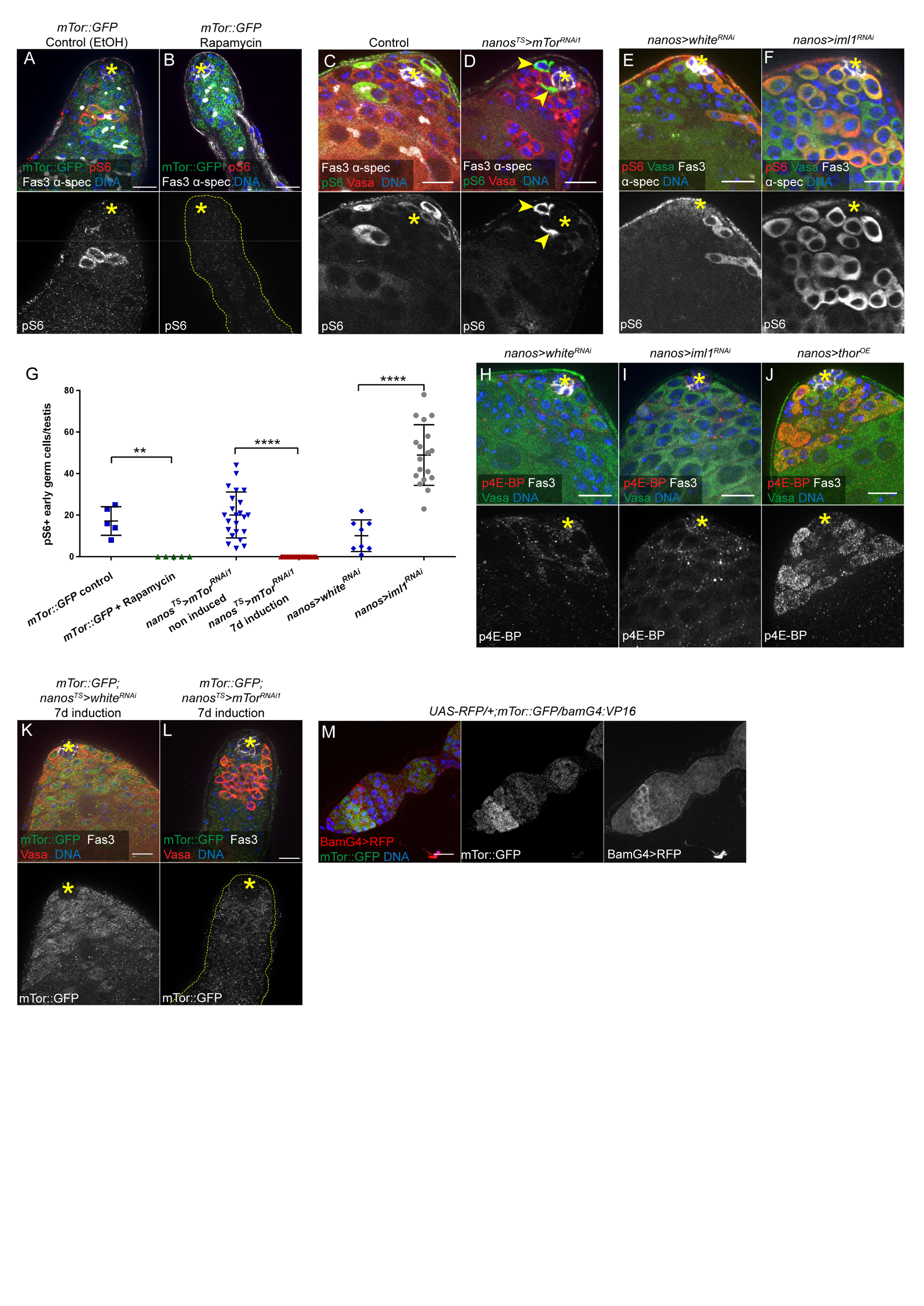

Supplement: S1 Fig — (A-B) Rapamycin treatment leads to a loss of pS6 staining without change in mTor::GFP expression. mTor::GFP flies were placed on food containing either ethanol as a control (A) or rapamycin (4mM in ethanol) (B) for 5 days. Images show testis tips with GFP, pS6, Fas3, α-spectrin and DNA stainings. Dotted lines delimitate the contour of the testis shown in (B). (C-D) RNAi-mediated depletion of mTor in germ cells induces a loss of pS6. Images show testis tips from nanos-GAL4/+;UAS-mTorTRiP-HMS00904/tub-GAL80ts animals raised at 18°C and either maintained at 18°C (C) (Control) or shifted to 29°C at the adult stage for 7 days prior to dissection (D) (nanosTS>mTorRNAi1), with pS6, Fas3, α-spectrin, Vasa and DNA stainings. Arrowheads on panel (D) signal Vasa- pS6+ cyst cells. (E-F) Genetically induced hyperactivation of mTORC1 leads to an increase in pS6-positive cells. Images show examples of testis tips from control (E) (nanos-GAL4:VP16/UAS-whiteTRiP-HMS00045) and Iml1-depleted (F) (UAS-iml1TRiP-HMC04806/+;nanos-GAL4:VP16/+) animals, with pS6, Vasa, Fas3, α-spectrin and DNA stainings. (G) Quantification of early germ cells with pS6 staining in the indicated conditions: mTor::GFP—control (n = 5), mTor::GFP + rapamycin (n = 5), nanosTS>mTorRNAi1 non induced (n = 24), nanosTS>mTorRNAi1 7 days induction (n = 27), nanos>whiteRNAi (n = 8), nanos>iml1RNAi (n = 18). ** (p<0.01) and **** (p<0.0001) denote statistical significance as determined with Mann-Whitney tests. (H-J) Phosphorylation of 4E-BP does not respond to variations in TOR activity in male germ cells. Images show testis tips from control (H) (nanos-GAL4:VP16/UAS-whiteTRiP-HMS00045), Iml1-depleted (I) (UAS-iml1TRiP-HMC04806/+;nanos-GAL4:VP16/+) and 4E-BP-overexpressing (J) (UAS-thor/+;nanos-GAL4:VP16/+), with p4E-BP, Fas3, Vasa and DNA stainings. (K-L) mTor::GFP expression strongly decreases upon downregulation of mTor in GSCs and early germ cells. Images show examples of testis tips from control (K) (nanos-GAL4/tub-G [file pone.0300337.s001.tif]

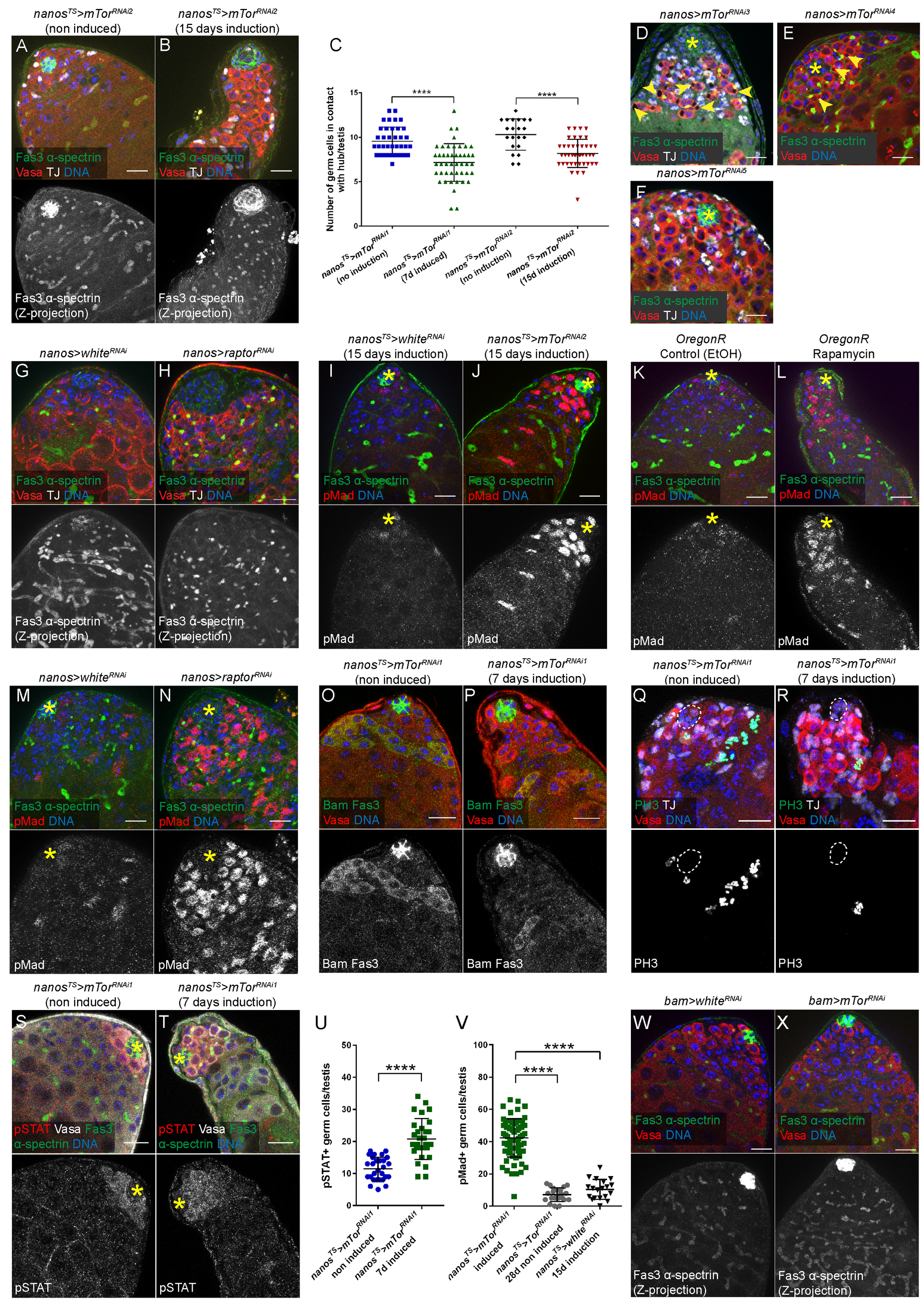

Supplement: S2 Fig — (A-B) Depletion of mTor in germ cells with mTorRNAi2 induces an accumulation of early germ cells with spherical fusomes. Images show testis tips from nanos-GAL4/+;UAS-mTorTRiP-HMS01114/tub-GAL80ts animals raised at 18°C and either maintained at 18°C (A) (Control) or shifted to 29°C at the adult stage for 15 days prior to dissection (B) (nanosTS>mTorRNAi2), with Vasa, α-spectrin (α-spec), Fas3, Traffic-Jam (TJ) and DNA stainings. Lower panels show maximum intensity projections of several images spanning the hub region (Z-projections). (C) Quantification of the number of germ cells (Vasa+ cells) in contact with the hub in control testes (nanosTS>mTorRNAi1—no induction, n = 39 and nanosTS>mTorRNAi2—no induction, n = 21) and in testes with mTor depletion in adult GSCs and early germ cells (nanosTS>mTorRNAi1 - 7d induced, n = 46 and nanosTS>mTorRNAi2 - 15d induced, n = 43). **** denotes statistical significance (p<0.0001) as determined with Mann-Whitney tests. (D-F) Germline phenotypes induced by mTor depletion in GSCs and early germ cells throughout development with different RNAi (mTorRNAi3, mTorRNAi4, mTorRNAi5). mTorRNAi3 induces an accumulation of GSC-like cells with spherical fusomes and large autolysosomes (arrowheads), concomitant with loss of differentiating spermatogonia (D). mTorRNAi4 causes a milder phenotype, with germ cells with spherical fusomes several cell diameters away from the hub and large autolysosomes (arrowheads, E). No obvious phenotype was observed with mTorRNAi5 in early germ cells (F). Testes are stained with Vasa, α-spectrin, Fas3, DAPI (DNA) (D-F) and Traffic Jam (TJ, in D and F). (G-H) RNAi-mediated depletion of Raptor in germ cells induces an accumulation of early germ cells with spherical fusomes. Images show examples of testis tips from control (G) (nanos-GAL4:VP16/UAS-whiteTRiP-HMS00045) and nanos-GAL4:VP16/UAS-raptorTRiP-HMS00124 (H) animals, with Vasa, α-spectrin (α-spec), Fas3, Traffic-Jam (TJ) and DNA stainings. Lower panels show ma [file pone.0300337.s002.tif]

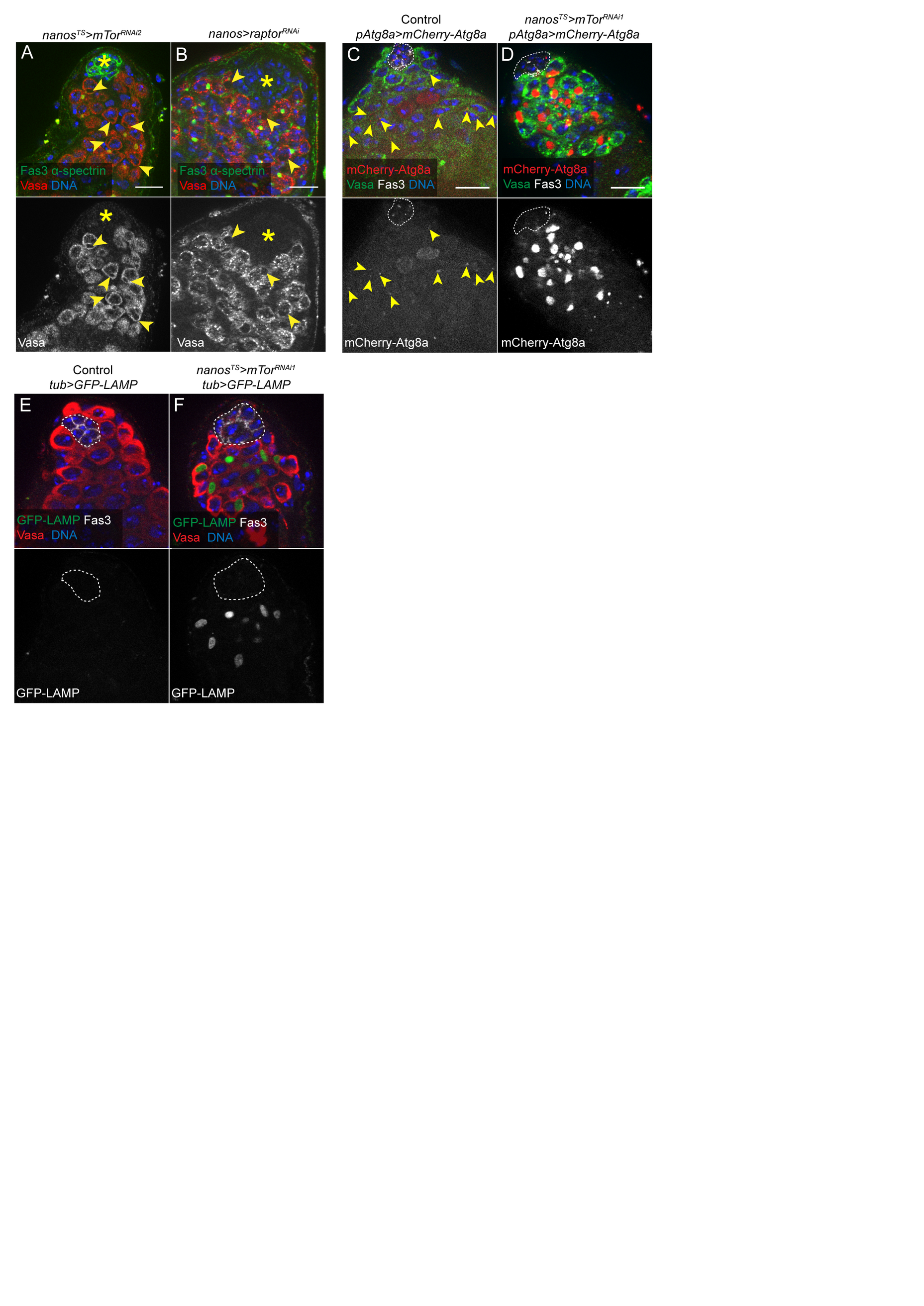

Supplement: S3 Fig — (A-B) mTor-depleted and Raptor-depleted germ cells harbor large cytoplasmic areas devoid of Vasa staining. Images show testis tips from nanos-GAL4/+;UAS-mTorTRiP-HMS01114/tub-GAL80ts animals raised at 18°C and shifted to 29°C at the adult stage for 15 days prior to dissection (A) and nanos-GAL4:VP16/UAS-raptorTRiP-HMS00124 (B) animals, with Fas3, α-spectrin, Vasa and DNA stainings. Arrowheads point to Vasa-negative structures in the cytoplasm of germ cells. (C-D) Vasa-negative regions in mTor-depleted germ cells contain Atg8a. Images show testis tips from pAtg8a-mCherry-Atg8a/nanos-GAL4;UAS-mTorTRiP-HMS00904/tub-GAL80ts animals raised at 18°C and either maintained at 18°C (C) (Control) or shifted to 29°C at the adult stage for 7 days prior to dissection (D), with Vasa, Fas3 and DNA stainings. Arrowheads in (C) point to Atg8a puncta in cyst cells and germ cells in a control testis. (E-F) GFP-LAMP accumulates in Vasa-negative cytoplasmic structures of mTor-depleted germ cells. Images show testis tips from tub-GFP::LAMP1/nanos-GAL4;UAS-mTorTRiP-HMS00904/tub-GAL80ts animals raised at 18°C and either maintained at 18°C (E) (Control) or shifted to 29°C at the adult stage for 7 days prior to dissection (F), with Vasa, Fas3 and DNA stainings. The hub is indicated with an asterisk in (A-B) and is delimitated with dotted lines in (C-F). For all images, scale bar: 15μm. (TIF) [file pone.0300337.s003.tif]

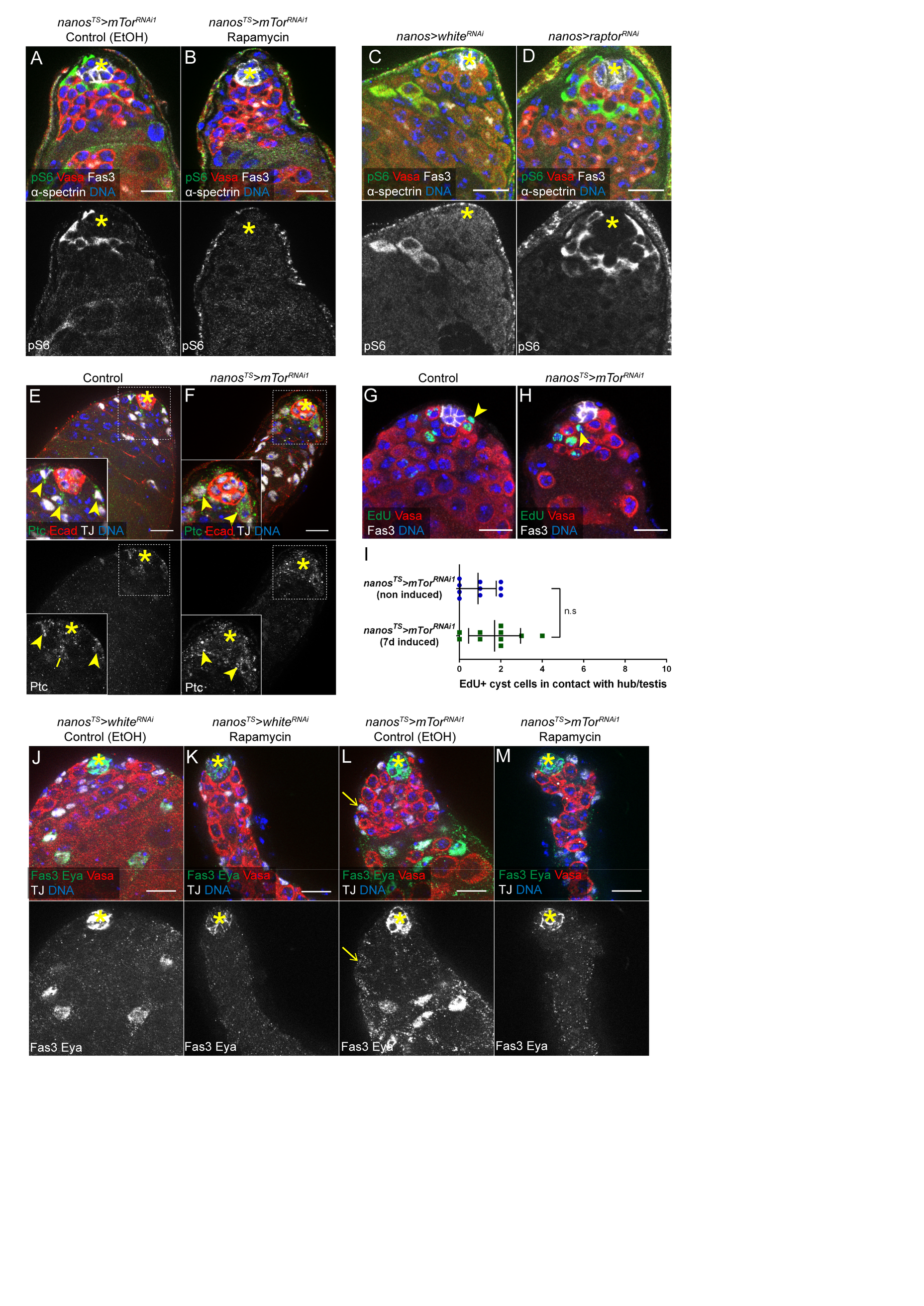

Supplement: S4 Fig — (A-B) Rapamycin treatment prevents the increase of pS6 in cyst cells adjacent to mTor-depleted germ cells. nanos-GAL4/+;UAS-mTorTRiP-HMS00904/tub-GAL80ts flies raised at 18°C were shifted to 29°C at the adult stage on food containing either ethanol as a control (A) or rapamycin (4mM in ethanol) (B) for 7 days. Images show testis tips with pS6, Vasa, Fas3, α-spectrin and DNA stainings. (C-D) Depletion of Raptor in germ cells induces an increase of pS6 in neighboring cyst cells. Images show testis tips from nanos-GAL4:VP16/UAS-whiteTRiP-HMS00045 (C) and nanos-GAL4:VP16/UAS-raptorTRiP-HMS00124 (D) animals, with pS6, Vasa, Fas3, α-spectrin and DNA stainings. Cyst cells are identified as Vasa-negative and Fas3-negative cells in the testis tip. (E-F) Depletion of mTor in adult germ cells does not affect Patched expression in cyst cells. Images show testis tips from nanos-GAL4/+;UAS-mTorTRiP-HMS00904/tub-GAL80ts animals raised at 18°C and either maintained at 18°C (E) or shifted to 29°C at the adult stage for 7 days prior to dissection (F), with Patched (Ptc), E-Cadherin (Ecad), TJ and DNA stainings. Arrowheads point to Ptc-positive and TJ-positive cells in contact to the hub, identified at CySCs. (G-I) Depletion of mTor in adult germ cells does not affect the ability of CySCs to proliferate. Images show testis tips from nanos-GAL4/+;UAS-mTorTRiP-HMS00904/tub-GAL80ts animals raised at 18°C and either maintained at 18°C (G) or shifted to 29°C at the adult stage for 7 days prior to dissection (H), with EdU, Vasa, Fas3 and DNA stainings. Arrowheads point to EdU-positive and Vasa-negative cells in contact to the hub, identified at CySCs. The graph in (I) shows the number of EdU positive CySCs per testis in the indicated conditions (n = 10 in each condition), “n.s” indicates that the numbers are not significantly different as determined with a Mann-Whitney test. (J-M) Treatment with Rapamycin inhibits Eya expression both in control testes and testes with germline-specific Tor d [file pone.0300337.s004.tif]
