# Supplementary material for: mTORC1 is required for differentiation of germline stem cells in the Drosophila melanogaster testis
Source: PLoS One. 2024 Mar 21;19(3):e0300337. doi: 10.1371/journal.pone.0300337 (PMC10956854; doi:10.1371/journal.pone.0300337)
Supplement: Attachment — Submitted filename: Response to Reviewers.0521.pdf [file pone.0300337.s005.pdf]

We thank the Reviewers for their feedback for our manuscript.  Please find a point-by-point response to their constructive criticism and suggestions below.

**Reviewer #1:**

In this manuscript, Clemot et al., found that Tor signaling is activated in male GSC and early germ cells. Knockdown (KD) tor in germ cells results in a delay/block of germ cell differentiation, causing accumulation of ectopic GSC-like cells accompanied with elevated BMP signaling and dysfunctional autolysosomes. Furthermore, KD tor in the germline also induces Tor activation in the somatic cells and that may lead to premature differentiation of cyst cells. These results suggest that Tor controls proper differentiation of germ cells in the male testis. Although the phenotypic observation tor KD germ cells is interesting, while molecular mechanisms are less addressed in the study.

Specific points

1. Since the author claims metabolism is important for germ cells, and mTOR regulates metabolism. Should not the author show how metabolism is altered in germ cells when Tor signaling is disrupted?

→ We show that disruption of TOR signaling results in the accumulation of dysfunctional autolysosomes and an accumulation of Ref(2)P, suggesting a defect in proteostasis.

How Tor signaling promotes germ cell differentiation? If increased BMP signaling is not the primary factor to suppress germ cell differentiation (as the author mentioned in the discussion), then what could be the mechanism?

→ In the Discussion, we note that : "Although we find that BMP signaling is upregulated upon mTORC1 inhibition in GSCs, hyperactivation of BMP signaling in GSCs is not sufficient to prevent the transition to TA divisions [42,43,67]. Therefore, this is not likely to be the primary mechanism underlying the block in differentiation in male germ cells."
We did attempt to downregulate BMP signaling concomitantly with mTor depletion in adult germ cells to test whether this would be sufficient to result the germ cell phenotype. However, none of the manipulations tested ($Tkv^{RNAi}$, $Mad^{RNAi}$ and $Medea^{RNAi}$) resulted in a decrease in $pMad^{+}$ cells, nor did it alleviate the block in differentiation. These findings indicated that these RNAi lines were not efficiently expressed with the $nanos^{TS}$ system, which we recognize constitutes an important limitation for our study, as discussed below. To avoid any confusion, we removed this section from the discussion.  Another possibility could be the accumulation of large autolysosomes that may sequester important factors limiting for differentiation.  For the same technical reason stated above, we were unable to determine whether manipulation of components of the autophagy machinery would suppress the germ cell phenotype.

Dysfunctional autophagosomes could be just the consequent results from Tor KD.

→ Absolutely- inhibition of TOR is known to induce autophagy. Although autophagy is upregulated, the resulting autophagolysosomes are unusually large and are not functional, which was unexpected.

How Tor KD activated BMP signaling? Is it because ligands are increased in the somatic cells?

→ We discuss that the regulation of BMP signaling by Tor is likely conserved, as it is observed in several other systems including hair follicle stem cells. However, the mechanisms remain unknown.
→ An increase in ligands in somatic cells, as proposed by the reviewer, is an interesting hypothesis. It is also possible that the turnover of Tkv may be affected by downregulation of TOR signaling and the ensuing perturbation in proteostasis.

How does KD tor in germ cells to increase tor signaling in the soma.

→ As mentioned in the discussion, one possibility is that metabolites released from germ cells, such as degradation products resulting from autophagy activation upon mTORC1 inhibition, act as signals to activate mTORC1 in neighboring cells.

To test whether upregulation of autophagy could be involved in the increase in TOR signaling in the soma, we combined *mTor*$^{RNAi}$ with either *Atg1*$^{RNAi}$ or *Atg8a*$^{RNAi}$. Unfortunately, as noted above, these RNAi lines did not efficiently downregulate autophagy when expressed under the control of *nanos*$^{TS}$, and these experiments were inconclusive. We also sought to investigate the contribution of lysosomal exocytosis and non-conventional secretion, by combining *mTor*$^{RNAi}$ with *rab27*$^{RNAi}$, *rab11*$^{DN}$ (UAS transgene) or *zpg*$^{RNAi}$, but were similarly limited by the variability in the efficiency of transgene expression with the *nanos*$^{TS}$ driver.

Does tor cross talk with Bam for germ cell differentiation?

→ High BMP signaling is expected to prevent Bam expression. Please see comment above regarding the possible involvement of elevated BMP signaling in the differentiation block. We do not know if there is a direct connection between Tor and Bam.

In short, there is no mechanism provided in this study.

→ Indeed, our study provides a descriptive analysis of the role of TOR in early germ cells, which had not been previously characterized. The highly variable efficiency of the *nanos*$^{TS}$ system to induce conditional transgene expression in adult GSCs and early germ cells is an important technical challenge that limited epistasis analysis and, consequently, any mechanistic insight. In the future, this tool must be improved in order to further investigate the mechanisms involved in the phenotypes we describe.

2. In addition to RNAi, can the author use GSC clone to trace tor mut GSC behavior?

→ Analysis of *mTor* mutant GSC clones would nicely complement our data. We attempted to generate *mTor*$^{\Delta 40}$ mutant GSC clones, using both a twin spot clone approach and MARCM.

- Using the twin spot clonal induction method, with a ubiquitously expressed marker that is lost in mutant clones, we were able to observe *mTor* mutant spermatogonial cysts, characterized by a small cell size, elevated Vasa expression and more condensed DNA compared to control twin clones (Panel A in the figure below). However, the markers available (such as UbiGFP), were not expressed in wild-type GSCs even without clonal induction, which prevented the distinction of clonal GSCs that have lost this marker (Panel B in figure below).

A.

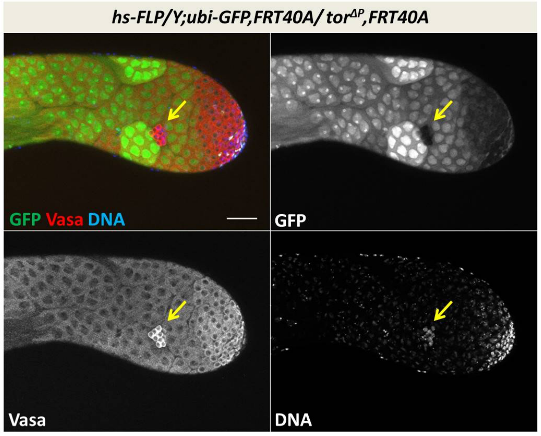


B.

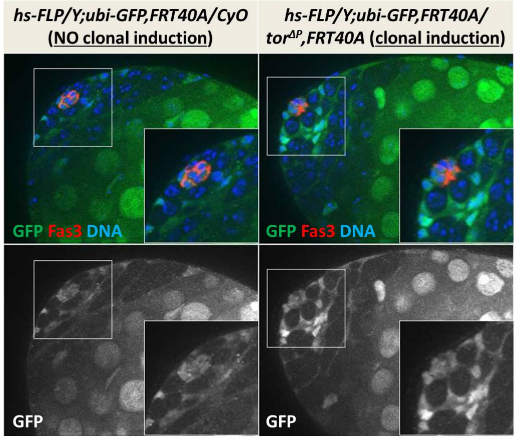


- Using MARCM, we were able to recover control clonal GSCs marked with GFP, but we never observed *mTor* mutant GSCs expressing GFP, even at the earliest time point after clonal induction (1day post heat-shock). Occasionally, *mTor* mutant spermatogonial cysts expressing GFP were observed (arrow in Panel C below). When a *UAS-mTor$^{WT}$* rescue transgene was co-expressed, *mTor* mutant CySC clones were rescued but we still did not observe any *mTor* mutant GSC clone.

C.

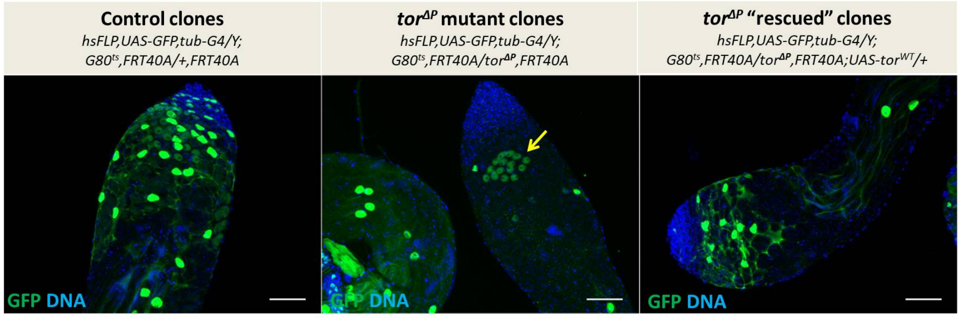


D.

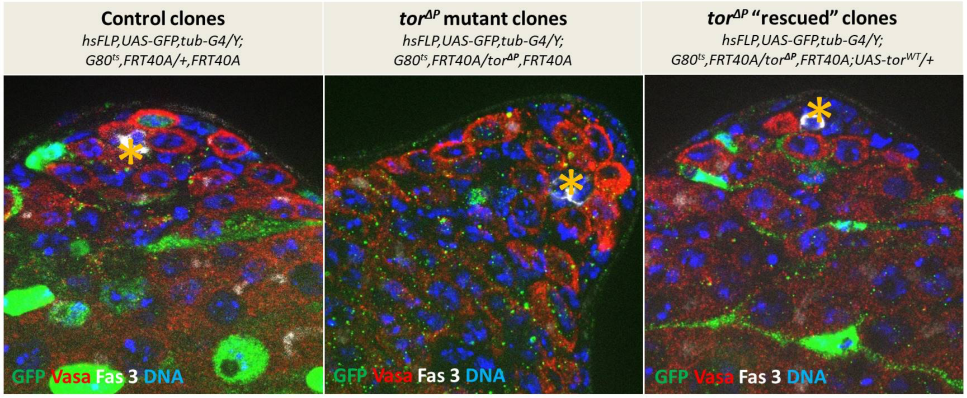


| Testes with GFP+ GSC(s): | 18/28 | 0/32 | 0/32 |
|---|---|---|---|

We are unsure how to interpret the absence of *mTor* mutant GFP+GSC at any time point. Given these difficulties that precluded the observation of mutant GSCs, we did not include these data in the manuscript and focused our analysis on the RNAi approach with multiple RNAi lines targeting different components of the pathway, which provided robust results.

3. In fig. 1D, some germ cells with extremely high pS6 expression, but all germ cells display similar levels of Tor::GFP. What does this mean? Are those germ cells with different tor activity under different status? Can the author provide quantification data?

→ Our data indicate that mTORC1 activity is patchy, consistent with what was shown in imaginal discs for example (Kim et al., 2017 and Romero-Pozuelo et al., 2017). While mTor::GFP expression is a pre-requisite, many signals regulate TOR signaling. We hypothesize that one possible factor is cell cycle status, as in imaginal discs.

→ We now provide a quantification of pS6+ germ cells in control testes and testes in which TOR signaling is either downregulated or upregulated (Supp. Fig 1J)

4. Does KD tor reduce GSC number? Any quantification data?

→ We now provide a quantification of GSCs, defined as Vasa positive cells in contact with the hub. Depletion of mTor with $nanos^{TS}$ leads to a modest, but significant, decrease in GSC number (described in the Results section lines 175-177 and shown in Supp. Fig 2C).

5. For fig.3 can the author provide quantification data? For example, penetration of the observed phenotypes, the size of autophagosomes, the number of tor KD germ cells carrying big autophagosomes? These germ cells die later or can differentiate later? Can tor KD testis generate sperm? are tor KD males sterile?

→ This phenotype is extremely penetrant, as all mTor-depleted testes examined harbored germ cells with large autolysosomes. We now provide quantification in Fig 3C (Results section lines 215-216).

→ As per the reviewer's suggestion, we measured the area of autolysosomes observed in mTor-depleted germ cells, compared to area of the autolysosomes seen in control germ cells (Fig 3H, Results section line 247). This confirmed that autolysosomes formed upon mTor depletion in germ cells are strikingly large compared to "normal" autolysosomes.

→ Males expressing mTor RNAi in adult GSCs and early germ cells progressively stop producing sperm and become sterile. This is now noted in line 174.

6. In Fig. 4, how if the author simply overexpress Tor in the somatic cells and to see it effects on cyst cell themselves and germ cell differentiation?

→ Published data has described the role of TOR signaling in differentiation of the cyst cell lineage (Amoyel et al., 2016, Yuen et al., 2021). These studies suggested that TOR signaling is required for cyst cell differentiation but is not sufficient, alone, to drive differentiation.

**Reviewer #2**:
TORC1 is a central regulator of metabolism in eukaryotes which promotes anabolism and inhibits catabolism. Tight control of metabolism is critical to stem cell maintenance as well as the differentiation of stem cell progeny in many systems. Here Clemot, Jones and colleagues, examine the role of TORC1 in the differentiation of the male germ line in Drosophila. They find that the expression of the serine/ threonine kinase Tor,is upregulated

in germline stem cells and their progeny. They go on to demonstrate that reducing Tor levels results in a block to germ cell differentiation and the accumulation of cells with features of germline stem cells. Additionally, they report that depleting Tor in the male germline results in a nonautonomous increase in TORC1 activity in somatic cyst cells. Overall, the manuscript is well written, and the author's conclusion are supported by their data. This work will be of interest to readers with an interest in germ cell biology, stem cells and metabolism. However, there are multiple issues that must be addressed.

Comments

1. In larval imaginal discs, pS6 levels oscillate with the cell cycle, with the highest levels observed in G1 and early S phase. Is it possible that they reason you see high pS6 levels in early germ cells simply reflects the fact that these cells are undergoing mitotic cell cycles while older germ cells do not?

→ Yes, it is indeed possible that differences in cell cycle status may explain the patchiness of mTORC1 signaling within the cells that express mTor::GFP, as it is the case in wing imaginal discs (Kim et al., 2017; Romero-Pozuelo at al., 2017).

2. The authors use a tagged Tor::GFP reporter to analyze endogenous Tor expression. Does this reporter construct rescue a Tor null allele? I suspect not, Tor is notoriously difficult to tag without killing protein function. Thus, while it might be fine to follow expression levels of Tor with this reagent, I am extremely skeptical of reports of the proteins intracellular localization without a demonstration that Tor::GFP is functional. On line 131 the authors report that Tor::GFP becomes nuclear in bam positive cells. The nuclear localization of Tor at a level that can be observed by immunofluorescence is an unusual, and thus potentially exciting observation. However, it is difficult to gauge the biological relevance of this result if the Tor::GFP protein is not functional.

→ It has been demonstrated previously that the *mTor::GFP* transgene does not fully rescue an *mTor* null allele (Sarov et al., 2016). Therefore, we can only use this tool to infer that mTor expression is not uniform in the germline, which is a novel observation. We agree with the reviewer that this reporter cannot be used to analyze the intracellular localization of mTor; therefore, we removed reference to the nuclear localization of mTor::GFP (line 135). We now state this limitation in the Material and Methods (Fly stocks and Genetics section).

3. Line 152: The authors state that their knockdown experiments of Tor demonstrate that "Tor activity is regulated in part by the differential expression of the Tor Kinase." I disagree, their experiments simply demonstrate that Tor protein is required for Tor activity. This is not surprising. The statement is not necessarily incorrect, it is just not proven by the experiments.

→This statement is not related to the mTor knock-down experiments that are described in the following section of the manuscript, but rather to the finding that mTor expression is not uniform. Since mTor expression is a pre-requisite for it to be active, differential expression of the kinase constitutes one layer of regulation of TOR activity. We found this interesting because this could explain the variability in TOR activity in other systems as well and reports of mTor expression are relatively scarce.

4. Line 193: The authors indicate that RNAi knockdowns of Tor do not produce phenotypes in bam expressing cells. It is inappropriate to make negative conclusions from

RNAi data where one does not know the extent of knockdown. At minimum the authors need to soften their language.

→ Given that there is little expression of mTor::GFP in *bamGAL4* expressing cells (as shown in Fig1) and that we do not observe pS6 in these cells, it is not surprising that expression of *mTor*$^{RNAi}$ with *bamGAL4* does not induce any obvious phenotype, according to the markers that we assessed (Vasa and α-spectrin). We simply state the results but do not go so far as to suggest that this means Tor has no role in these cells.

5. Line 202: The authors demonstrate that Tor depletion results in the accumulation of large dysfunctional autolysosomes in the early male germline. The authors should note that the reactivation of Tor is required for the reformation of lysosomes from autolysosomes (Nature. 2010 Jun 17; 465(7300): 942–946.). Thus, depleting Tor is predicted to block the process of lysosome reformation. In the presence of Tor, breakdown products from autophagy allow for the reactivation of TORC1 and the reformation of lysosomes. This process would be blocked in the absence of Tor.

→ This is indeed an interesting process that we mentioned in the Discussion as one possible explanation for the dysfunctional autophagy/lysosomal degradation, as well as a potential mechanism through which TOR could be activated in neighboring cyst cells by possible degradation products. This reference is cited in the Discussion (line 370).

6. Throughout the manuscript the figure labels are difficult to read because they are too small. Additionally, the labels often blend into the background of the image. These issues will be amplified when the figures are reduced in size for publication. Many of the dashed lines are also extremely thin and difficult to see, in panel 1B for example.

→ We have now increased the size of the labels in all figures.

7. The RFP reporter is not in the key in Figure 1B.

→ This is now corrected.

**Reviewer #3**:

<u>Summary</u>

Target of Rapamycin (TOR) is a major nutrient sensor that regulates many cellular processes, including growth, proliferation, metabolism, and autophagy. Previous studies by many groups have shown that TOR is a major regulator of various aspects of stem cell lineages (e.g. stem cell proliferation and maintenance, survival, growth, and differentiation of their progeny, etc) across many stem cell systems, including Drosophila female germline stem cell, intestinal stem cell, ovarian and testicular somatic stem cell lineages, C. elegans germline, and mammalian intestinal stem cells, neural stem cells, hematopoietic stem cells, muscle stem cells, and basal cells in the airway. In this study, Clémot et al. use a TOR::GFP fusion, RNAi-based approaches, and signaling and autophagy reporters to investigate the role of Tor in the male germline stem cell lineage. They report that Tor function is required in the germline for differentiation of spermatogonia (i.e. early germ cells) and, non-cell autonomously, for preventing the premature differentiation of somatic cyst cells surrounding germ cells. They also report that knocking down germline TOR signaling increases BMP signaling in germ cells (known to be required for germline stem cell maintenance/inhibition of differentiation) and leads to formation of large and defective autolysosomes.

Critique

The manuscript is well written overall and describes new roles for TOR signaling in early germ cells in the Drosophila testis. The novelty of the study is limited given the large number of studies showing that TOR has many complex roles across a wide range of stem cell lineages. There are also significant rigor/reproducibility concerns because the authors do not consistently include proper controls and data quantification. In addition, the authors did not perform straightforward and easily feasible (in Drosophila) follow up experiments to address simple questions/models that their correlative observations raise.

Major Points:

1) To examine the expression pattern of TOR, the authors used a publicly available strain expressing a TOR::GFP fusion protein. The Tor::GFP transgene represents a large genomic region including Tor with a GFP tag at the C-terminus (plus regulatory regions), and it is designed for the TOR::GFP fusion to reflect endogenous expression. However, the authors did not test if the Tor::GFP transgene rescues the lethality of homozygous null Tor mutants, which would be important to validate that TOR::GFP indeed behaves like the endogenous TOR protein. The authors should explicitly discuss these caveats.

→ As mentioned in response to Reviewer 2's comment above, it has been shown previously that the *mTor::GFP* transgene does not fully rescue a *mTor* null allele (Sarov et al., 2016). Therefore, we use this tool only to determine that mTor expression is not uniform in the germline, which is a novel observation. As this reporter cannot be used to analyze the intracellular localization of mTor, we removed reference to the nuclear localization of mTor::GFP in the Results section. In addition, we now discuss this limitation in the Material and Methods (Fly stocks and Genetics section).

2) In this study, only one Tor RNAi line is used for all experiments (except for one instance where a second Tor RNAi line was used but analyzed at a later time point; see Point 3 below) – and there is no measurement of RNAi knockdown efficiency. Also, only one Raptor RNAi line is used.

→ Five RNAi transgenes targeting different regions of *mTor* mRNA were tested (listed in Table 1). *mTor*$^{RNAi1}$, *mTor*$^{RNAi2}$ and *mTor*$^{RNAi3}$ caused the strongest phenotypes when expressed in germ cells throughout development with *nanosGAL4:VP16*. *mTor*$^{RNAi4}$ caused a milder phenotype and *mTor*$^{RNAi5}$ did not cause any obvious phenotype. Thus, we selected *mTor*$^{RNAi1}$, *mTor*$^{RNAi2}$ and *mTor*$^{RNAi3}$ for expression in adult germ cells using *nanos*$^{TS}$. *mTor*$^{RNAi1}$ and *mTor*$^{RNAi2}$ robustly induced the same phenotype, together with an efficient reduction in mTORC1 activity, assessed by pS6 staining after 7 days (*mTor*$^{RNAi1}$) and 15 days of induction at 29°C (*mTor*$^{RNAi2}$). By contrast, *mTor*$^{RNAi3}$ did not result in efficient reduction of pS6, even after 15 days of induction, illustrating the limitations of the *nanos*$^{TS}$ system (please see response to Reviewer 1 above). Therefore, we chose to present the data obtained with *mTor*$^{RNAi1}$, the most efficient RNAi line, in the main figures and included data obtained with *mTor*$^{RNAi2}$ to support these results in the Supplementary Figures. We now detailed our approach in the Material and Methods section (in the Fly Stocks and Genetics subsection) and we included representative images for the all five RNAi lines in Supplementary Figure 2.

In support of these data, we also show that systemic inhibition of mTORC1 signaling in adult flies causes a block in germ cell differentiation, with an accumulation of early germ cells with spherical fusomes and high pMad. Moreover, *raptor*$^{RNAi}$ was used as an additional

confirmation that the observed phenotypes are caused by mTORC1 downregulation. As with the TorRNAi lines, we tested several RNAi lines and selected the one with which we observed the most efficient loss of pS6.

There are many available transgenes to manipulate TOR activity in Drosophila, including for non RNAi approaches; however, the authors did not take advantage of these resources.

→ We are not sure which transgenes to which the reviewer is referring. We tested multiple approaches available to us to manipulate TOR activity, including the generation of mutant clones, which proved to be inconclusive in our system (see above). We preferred the RNAi based approach because it was the most efficient, despite its limitations, and best suited for our objective to specifically deplete mTor from adult GSCs and early germ cells.

The use of one Tor RNAi line for most experiments in the manuscript decreases the rigor of the experiments (e.g. potential off-target effects are not effectively ruled out).

→ Please see details of our RNAi approach above. Multiple *mTor*$^{RNAi}$ lines targeting different regions of the mRNA, as well as RNAi of another component of the pathway, Raptor, and systemic inhibition of mTor with Rapamycin all induce the same phenotypes. In our opinion, this strongly argues that these are unlikely due to off-target effects. Information regarding the lines used are in Table 1.

3) The vast majority of Tor RNAi experiments involving a shift from 18oC to 29oC in this study lack the appropriate control for temperature (29oC) and engagement of the RNAi machinery, such as nanos>control RNAi (e.g. white RNAi) shifted to 29oc in parallel to experimental genotypes. The nos>Tor RNAi uninduced control is far from ideal (18oC temperature and no engagement of the RNAi machinery). In lines 190-193 (Fig S2I), the authors attempt to address this issue specifically for the experiment showing accumulation of pMad+ cells; however, the “experimental” (nos>TorRNAi) and “control” (nanos>white RNAi) shown in Fig. S2I are from separate experiments (i.e. nos>Tor RNAi data are from the experiment shown in Fig. 2G, as the authors themselves point out) and induced at 29oC for different number of days (7 days for Tor RNAi versus 10-15 days[?] for white RNAi).

→ Given our observation that the *nanos*$^{TS}$ system had variable efficiency depending on UAS transgenes, as mentioned above, we decided that flies from the same genotype but kept at 18°C (non-induced) were the most appropriate control for our RNAi experiments. To verify that the observed phenotypes were not a result of engagement of the RNAi machinery itself, we included data showing that *nanos*$^{TS}$>*white*$^{RNAi}$ induced at 29°C did not present any phenotype (Supplementary Figures 1A, 2G, 2M and 4G). As the reviewer correctly notes, the induction time for *nanos*$^{TS}$>*white*$^{RNAi}$ varies between the different figures, to match the specific experiment. For example, in the case of pMad stainings, *nanos*$^{TS}$>*white*$^{RNAi}$ was analyzed in parallel with *nanos*$^{TS}$>*mTor*$^{RNAi2}$, which requires a longer induction time (as explained above and in the Material and Methods). We reasoned that if there was no phenotype after 15 days of induction at 29°C, there was also likely no phenotype after shorter induction times. In addition, we show that the effects we observed are not due to accelerated aging of the flies at 29°C, since after 4weeks at 18°C there is no phenotype (quantification of pMad-positive germ cells is shown in Supplementary Fig 2M).

4) With the exception of experiments in Fig. 2G, S2I and F, experiments in this study were not quantified, and the authors do not mention specifically how many times each experiment was independently performed, or what the samples sizes were within each replicate.

→ In addition to quantification of pMad$^+$ germ cells (Figure 2G, Supp. Figure 2M), pS6$^+$ somatic cells (Figure 3C) and TJ$^+$/Eya$^-$ cells (Figure 3F), we now provide quantification of pS6$^+$ early germ cells (Supp. Figure 1J), GSC number (Supp. Figure 2C), number or autolysosomes in germ cells (Figure 3C) and autophagolysosomes area (Figure 3F).

→ All images shown are representative images of at least three biological replicates (independent crosses), with 20 to 24 testes per replicate. We have now stated this clearly in the Materials and Methods and indicated sample sizes (n) for quantifications in the figure legends.

5) Page 6-7 & Figure 1: Related to point 1 above, it remains unclear how well the TOR::GFP pattern reflects that of endogenous TOR, especially considering that its levels of expression do not correlate well with the levels of TOR activity detected by pS6 antibodies. For example, in Fig. 1D, the relative intensity of TOR::GFP (in both outlined and non-outlined germ cells) does not correlate with the relative intensity of pS6 – this is inconsistent with the authors' conclusion that "TOR activity is regulated at least in part through differential expression of the Tor kinase" (line 153). These discrepancies should be addressed in the Results and Discussion sections. The data presented in Figure 1 (and S1) are not quantified, and the sample sizes analyzed (or how consistent the staining was from testis to testis) are not mentioned.

→ The use of the mTor::GFP transgene allows us to report that mTor expression is not uniform. Since mTor expression is a pre-requisite for it to be active, differential expression of the kinase constitutes one layer of regulation of TOR activity. The variability of the pS6 signal within mTor-expressing cells suggests that (not surprisingly) other cues are involved in regulating TOR activity. This "patchiness" of TOR activity has been described in other tissues, such as the wing imaginal discs, in which cell cycle status was shown to be an important regulator of TOR activity and S6 phosphorylation (Kim et al., 2017; Romero-Pozuelo et al., 2017). Our observation that mTor is not uniformly expressed suggests that not all germ cells are equally permissive to such signals regulating TOR activity. We now provide a quantification of pS6+ early germ cells in Supplementary Figure 1J, which reflects the patchiness of TOR activity.

6) Figure 1D compared to Figure S1F: the (non-quantified) data presented in these panels suggest that expression of the TOR::GFP transgene alters the levels of TOR activity in the testis – note the higher levels of pS6 when TOR::GFP is present (1D) than when it is absent (S1F). This could potentially be due to different stability of the TOR::GFP fusion protein and/or the increased dosage of Tor by addition of this genomic construct to the two endogenous copies of Tor. Along those lines, p4E-BP is only tested when TOR::GFP is absent (S1H). These potential issues should be clearly discussed.

→ We thank the Reviewer for noting this important point.  Even if the mTor::GFP transgene does not fully rescue the phenotypes caused by a mTor null allele (Sarov et al., 2016), suggesting that the tagged protein is not fully functional, it could potentially increase the dosage of mTor protein and affect TOR pathway activity. However, we observed that pS6 staining in early germ cells was variable regardless of the presence of mTor::GFP. For example, the testes in Figure 1D and Supp Figure S1D both have the mTor::GFP transgene and show variability in the number of pS6+ germ cells. Control testes without mTor::GFP show a similar variability (please compare images in Supp. Figure 1F, Supp. Figure 1H and Figure 4A). We also did not see any difference in p4E-BP staining in testes with and without

mTor::GFP. Therefore, the mTor::GFP transgene does not appear to significantly affect the levels of TOR activity. We now provide quantification in Supplementary Figure 1J.

7) Lines 167-168 & Figure 2D: the authors mention in passing that "germ cells were still present at the tip of the testis in contact with the hub". The image suggests, however, that many of the "bona fide" germline stem cells (i.e. those in contact with the hub) are lost. The authors should address this point with appropriate quantification, as it might affect their conclusions about the role of TOR in self-renewal versus differentiation decisions in the testis germline (i.e. perhaps more complex than the interpretations in the manuscript suggest).

→ We now provide quantification of the number of GSCs, defined as Vasa positive germ cells in direct contact with the hub (Supplementary Figure 2C, Results section lines 175-177). Depletion of mTor depletion causes a modest, but significant, decrease in GSCs. However, many germ cells away from the hub harbor high pMad, another marker of GSC identity, making this result difficult to interpret. The best approach to investigate the role of TOR in self-renewal would be clonal analysis. However, as noted above in response to Reviewer 1, we were not able to recover *mTor* mutant GSC at any time point, which prevented us for making any reliable conclusions on the role of TOR in GSC self-renewal. By contrast, our data show that TOR is required for early germ cell differentiation.

8) Pages 12-13 & Figure 4/S4: in addition to the issues mentioned above (Points 2 and 3), there is a concern that by the timepoint when the testes are dissected and stained for pS6, the testis structure is altered, making it difficult to draw a direct connection between Tor RNAi in the germline and activation of TOR in cyst cells (see Fig. 4B; i.e. Are these secondary effects due to tissue abnormality?).

→ We are not aware of examples of tissue abnormality indirectly causing changes in TOR activity or other signaling pathway activity. The stainings shown in Figure 4B and quantified in Figure 4C were dissected and stained after 7days of induction at 29°C, the same time point that was used for the other experiments in our study. In addition, we observed the same ectopic TOR activity in cyst cells upon depletion of mTor in germ cells with $mTor^{RNAi2}$ and upon depletion of raptor in germ cells, which did not alter the testis structure as dramatically (Supp. Figure 4D). Furthermore, we show that this non-autonomous pS6 signal in cyst cells is indeed TOR-mediated, as it is lost upon rapamycin treatment, despite a similar effect on testis morphology as depletion of mTor in germ cells with $mTor^{RNAi1}$ (Supp. Figure 4B).

In addition, the authors should consider simultaneously knocking down Tor in the germline and cyst cells to test if premature induction of Eya is suppressed.

→ It is technically challenging to simultaneously knock down mTor in adult germ and cyst cells with a thermo-sensitive system. Expression of $mTor^{RNAi}$ in cyst cells only was previously shown to prevent Eya expression and cyst cell differentiation (Amoyel et al., 2016). Instead, we used systemic inhibition of mTORC1 by Rapamycin treatment to simultaneously downregulate the pathway in both cyst cells and germ cells. This resulted in a complete loss of pS6 and suppressed the premature induction of Eya expression observed upon germ cell-restricted downregulation of mTORC1 (shown in Supp. Figure 4J).

Another potential concern is that differentiation of cyst cells is assessed using a single marker (Eya), so it is difficult to distinguish if these results reflect changes in Eya expression

alone or in the entire cyst cell differentiation state. These potential caveats need to be incorporated into their conclusions both in Results and Discussion sections.

→ We observed both a reduction in the number early cyst cells (TJ+/Eya-) and the presence of Eya expression in somatic cells close to the hub upon mTor knock down in adult germ cells (Figure 4D-F). In addition, the nuclei of cyst cells, marked by TJ or Eya, appear larger in testes with mTor knock-down in germ cells. Together, these data suggest that cyst cells prematurely differentiate. However, we agree with the reviewer that it is difficult to distinguish between a change in Eya expression alone or in the entire cyst cell differentiation state, as Eya expression alone may not be sufficient to induce cyst cell differentiation (Fabrizio et al., 2003). We now note this in line 298.

9) Discussion, lines 335-344 & 352-354: the authors raise the possibility that the unusually large, defective autolysosomes present in Tor RNAi germ cells could be due to a combined effect of decreased TOR activity and increased BMP signaling. This would be easy to test by simultaneously knocking down Tor and Mad (for example) in the germline.
The authors also hypothesize that autophagy might be actively repressed by TOR signaling under homeostatic conditions (lines 343-344) and that activation of autophagy by Tor RNAi in germ cells might lead to activation of TOR in cyst cells (lines 352-354). It would be relevant and straightforward to test this model and how it relates to the Tor RNAi germ cell differentiation defect (e.g. double knockdown of Tor and Atg genes in germ cells [with proper controls] to measure autophagy markers in germ cells, how well germ cells differentiate, and TOR activation/differentiation markers in somatic cyst cells). These experiments, along with the experiment suggested in Point 8 above (simultaneous Tor RNAi in germ cells and cyst cells), are very feasible using routine fly genetics and microscopy and would allow the authors to directly test the functional relevance of some of the key correlative observations in this study.

→ We thank the reviewer for these suggestions and agree that such experiments, if conclusive, would indeed be of great interest to probe the mechanisms involved in the phenotypes we described. As mentioned in response to Reviewer 1, we attempted to simultaneously knock-down mTor and components of the BMP pathway (Tkv, Mad and Medea) in adult germ cells, in order to assess the contribution of high BMP signaling to the observed phenotypes. Unfortunately, none of the RNAi tested successfully lead to a downregulation of BMP signaling when expressed with *nanos*$^{TS}$.

To test whether upregulation of autophagy could be involved in the increase in TOR signaling in the soma, we combined *mTor*$^{RNAi}$ with either *Atg1*$^{RNAi}$ or *Atg8a*$^{RNAi}$, but these RNAi lines did not appear to downregulate autophagy when expressed under the control of *nanos*$^{TS}$ ; therefore, as with the BMP epistasis experiments, these experiments were inconclusive. We also sought to investigate the contribution of lysosomal exocytosis and non-conventional secretion (with *rab27*$^{RNAi}$, or *UAS-rab11*$^{DN}$ for example) but were similarly limited by the variability in the efficiency of transgene expression with the *nanos*$^{TS}$ driver. The technical challenges associated with the use of the *nanos*$^{TS}$ driver, now described in more details in the Material and Methods, significantly hindered our ability to test potential mechanisms and, in the future, this tool will need to be improved.

Additional Points:

- Figure 1 title: instead of “progenitor”, the authors should write “spermatogonial”; the word “progenitor” is confusing (and potentially misleading) in the context of the fly testis.

→ The updated title of Figure 1 in the revised manuscript is: “mTor expression and activity are higher in GSCs and early cells”.

- Line 125: Fig. 1B has no arrowhead nor arrow (both are mentioned in the text)
→ This is corrected in the revised version.

- Lines 128-130: it is not obvious how similar the TOR::GFP pattern is in the ovary and testis. The relevance of these data is also unclear, given that Tor is known to be required throughout oogenesis for different processes, and not just where TOR::GFP expression is readily detectable.

→ We analyzed mTor::GFP expression pattern in the first stages of oogenesis and found higher mTor expression in GSCs and early germ cells. To our knowledge, this appears consistent with published data on the role of mTORC1 in early oogenesis (LaFever et al., 2010; Sun et al.,2010).

- Line 130: not clear what regions 1 and 2a are based on text.

→ Regions 1 and 2a correspond to well-defined organization of the germarium, as per the reference cited (Spradling, 1993).

- Line 131: a brief explanation about bam and why it is relevant in this experiment should be provided for the readers’ benefit.
→ We have now added a brief description of Bam (line 136).

- Lines 160-162: in the results, the authors mention that the nanos-Gal4::VP16 driver was combined with Gal80 ts; however, this is not what the methods describe, which is confusing. On a related note, Gal80 does not bind to the Gal4::VP16 fusion, so Gal80 ts is not expected to have an effect on these modified versions of Gal4.

→ There is no mention of *nanos-GAL4:VP16*, which is not used in combination with $GAL80^{ts}$ (for the reason cited by the reviewer). By contrast, *nanos-GAL4:VP16* is used to express transgenes in early germ cells throughout development. We apologize for any confusion.

In lines 160-162, we provide a description of the $nanos^{TS}$ driver: “The *nanos-GAL4* ‘driver’ promotes expression in GSCs and early germ cells [34]; when combined with a ubiquitously expressed thermo-sensitive $GAL80^{TS}$ allele [35] (hereafter referred to as $nanos^{TS}$), this system permits depletion specifically in adult GSCs and early germ cells upon a shift to 29°C (cf. Methods).”

- Line 164: should say Supp. Fig 1B (not C)
- Line 189: should say Supp Fig 2G,H (not F,G)
- Line 228: should say Supp. Fig 3D (not F)
→ The figure calls have been corrected and updated in the revised manuscript.

- Fig 2 legend: arrowheads are mentioned, but Fig 2 has no arrowheads.
- Fig S2 legend: Delete the “pS6.” at the beginning of legend. Description of graph in (I) seems to include extra text that does not belong with this legend. Corrections also needed for 4th to last line of legend; J instead of G, K instead of H, and UAS-Tor instead of UAS-Raptor.

→ The figure legends have been corrected and updated in the revised manuscript.

- Line 432: remove “let”

→ Done

- Lines 458-459: the authors briefly state in the materials & methods that “all images shown are representative images of at least three independent experiments.” This is an insufficient demonstration of rigor and data robustness (see Point 4 above).

→ Please see response to Point 4.

- Table 1: The source of the UAS-RFP line is listed as “this publication”; however, the materials & methods have no information about how the authors generated this strain.

→ We have now included a description of the method used to generate this strain in the Materials and Methods (Page 19, lines 439-444).

- Table 1: Two tub-Gal80ts lines are listed, but unclear which one was used.

→ The two transgenes listed are located on different chromosomes (one is on chr II and one is on chr III). Both of them were combined with other transgenes and used depending on the chromosomal location of the other transgenes in the combination.

- Table 1: For all fly strains, should list specific genotypes/transgenes because the stock center numbers can change over time, potentially making it extremely challenging to figure out which strains were used in this study.

→ We have listed the specific transgene names and identifiers, in addition to the stock center numbers.

- Table 1: For rat monoclonal anti-Vasa, include clone name.

→ There is no clone name listed in DSHB (https://dshb.biology.uiowa.edu/anti-vasa).

- Table 1: Two rabbit polyclonals anti-phospho-dRpS6 are listed, but there is no information in the manuscript about which one was used for what experiment.

→ The two antibodies were produced in different labs using almost identical protocols (see Kim et al., 2017 and Romero-Pozuelo et al., 2017). We tested both in the testis and observed similar pattern. Therefore, they were used interchangeably in the experiments presented in the manuscript. We clarified this point in the Materials and Methods.
