# Supplementary material for: mTORC1 is required for differentiation of germline stem cells in the Drosophila melanogaster testis
Source: PLoS One. 2024 Mar 21;19(3):e0300337. doi: 10.1371/journal.pone.0300337 (PMC10956854; doi:10.1371/journal.pone.0300337)
Supplement: Attachment — Submitted filename: Response to Reviewers.0923.docx [file pone.0300337.s006.docx]

We thank the reviewers for their time and thoughtful comments that helped improve the manuscript. Please see our responses in blue font after each comment. Changes to the manuscript made in response to the reviewers’ comments are highlighted in yellow in the marked up, revised manuscript.

**Reviewer #1:**

In this manuscript, Clemot et al. describe analysis of TORC1 activity and function in male germline stem cells (GSCs) and early transit amplifying cells, primarily using a GFP-tagged mTor reporter and RNAi knockdown of TORC1 components and regulators. The authors describe their observations that variable TORC1 expression within these cells, which appears to be concentrated in the earliest germ cells, and that inhibition of TORC1 function in these cells disrupts germ cell progression and autolysosomal activity. The authors identify crosstalk between germ cells and somatic cyst cells that increases TORC1 activity in cyst cells when mTor is inhibited in the germline, which accelerates cyst cell differentiation.

While these observations are interesting and indicate that TORC1 activity is important within these cells, the major findings that TORC1 activity is restricted to the early germ cells and that downregulation of mTor in early germ cells causes a block / delay in differentiation are not fully supported by the data. The major concerns around these points are listed below:

1. The authors use a single reporter of mTor expression, a GFP-tagged mTor transgene. However, as the authors state, this transgene is unable to fully rescue mTor loss of function, suggesting insufficient function. It is unclear why the transgene does not fully rescue, and hence it is unclear whether such a reporter is fully recapitulating normal expression patterns. Because the high expression of mTOR in early germ cells serves as the foundational premise of this paper, additional validation of mTOR expression pattern is necessary. RNA in situ to examine mTOR expression might serve this purpose. Also multiple scRNA seq data of the testis are now available in the field, thus just reanalyzing them to see if mTOR is indeed concentrated in the early germ cells might suffice.

Our motivation to investigate the role of mTor in adult male GSCs primarily came from a previous study by our team, which found that mTor was activated upon perturbation of mitochondrial function in GSCs and that this was associated with precocious differentiation (Sênos Demarco et al., 2019). With the aim to study the role of mTor in GSCs, we first intended to gain insights into its expression pattern. Despite numerous studies on the TOR pathway, the expression of mTor itself is surprisingly rarely addressed and there were no available data in the testes at that time. Given the lack of suitable antibody against *Drosophila* mTor, we took advantage of the existing mTor-GFP transgene. We found particularly interesting the observation that mTor-GFP is not uniformly expressed in germ cells. We acknowledged that this may not entirely reflect mTor endogenous expression, as this transgene does not rescue lethality caused by a null allele and, therefore, we included a disclaimer in the initial submission. While this data is novel and supports our study, it is not meant to serve as the foundational premise of our work.

As suggested, we analyzed *mTor* RNA expression levels in the germline from the available Fly Cell Atlas scRNA-Seq data (Li H., Janssens J. et al., Science, 2022), using the Seurat R package. Average expression in each of the four annotated clusters (spermatogonium, mid-late proliferating spermatogonia, spermatogonium-spermatocyte transition and spermatocytes) indicates that average mTor RNA levels decrease as germ cells enter the spermatocyte stage (see Dot Plot below). This correlates with the decrease in mTor-GFP expression that we observe in spermatocytes (see Fig 1C for example). For comparison, we also examined expression of Thor mRNA in the same clusters. The Fly Cell Atlas data indicate that it is indeed expressed at very low levels in early germ cells/ spermatogonia, as suggested by our p4EBP stainings. We now reference the Fly Cell Atlas mRNA expression data in the revised manuscript lines 235-237 and line 283.

**Dot Plot graphical representation showing the expression pattern of mTor and Thor in germ cells, generated using the Fly Cell Atlas scRNA-Seq data (Li H., Janssens J. et al., Science, 2022).**

2. The authors use pS6 immunofluorescence as a readout of TORC1 activity, and have very strong controls to confirm this pS6 signal is dependent on TORC1 function (Rapamycin feeding and Iml1 RNAi). However, they observe pS6 sporadically throughout the early germline, it is not correlated with the strength of mTor-GFP, and is detected in both GSCs and transit amplifying cells (possibly as late as 8-cell spermatogonia). While the quantification of pS6 positive germ cells is useful, lumping together all “early germ cells” in this data obscures interpretation, particularly because it is unclear what stages this data includes (GSCs alone? GSCs through gonialblasts? Through 2-, or 4-cell spermatogonia?). More useful would be to separate this data into each stage of transit amplifying divisions to demonstrate both that pS6 is enriched in the earliest cells and that the reduction of pS6 upon mTor RNAi is specific to the earliest cells.

We did not intend to imply that mTor is most active in GSCs. Instead, we report that TORC1 activity is patchy in early germ cells (referring to nanos-expressing cells), consistent with what was shown in other tissues such as imaginal discs (Kim et al., 2017 and Romero-Pozuelo et al., 2017). As pointed out by the reviewer, the pS6 signal is not correlated with the strength of mTor-GFP and not all cells that express mTor-GFP have pS6. This is not surprising, as although expression of mTor is a pre-requisite for TORC1 activity, other signals (that could be activating or inhibiting) are at play. Other pathways that contribute to TORC1 activation (such as InR for example) will be the focus of further investigation.

As suggested by the reviewer and shown below, we classified the number of pS6 positive cells according to differentiation stage in wild-type testes (this cannot be done in testes expressing *mTor^RNAi^* under the control of *nanos^TS^*, as we observed a complete loss of pS6+ in germ cells in this background). This classification shows a trend in which pS6 is enriched in the earlier germ cell differentiation stages. However, we want to reiterate that we did not claim in the text that TORC1 activity is higher in one stage versus another in the *nanos-GAL4* expressing cells (see line 300).

3. The authors attempt to use phosphorylation of 4E-BP as a downstream readout of TORC1 activity, and are successfully able to do so through over expressing 4E-BP to make itself an available target for phosphorylation. However, the selected image in Supplementary Figure 1M demonstrates p4E-BP is almost exclusively in the most differentiated nanos expressing cells (where 4E-BP is being ectopically expressed), and is absent from the GSCs. This result undermines the authors’ claims that TORC1 is most active in GSCs. Again, full quantification of the % of p4E-BP cells among each cell type would strengthen the authors claims. Inclusion of Bam>thor over expression (and the expected lack of p4E-BP) would also help to strengthen this point. The authors do mention that there may be differential S6 and 4E-BP phosphorylation by TORC1, but they do not discuss the apparent more active 4E-BP phosphorylation in more differentiated cells or provide speculation as to what may cause this effect.

As clarified above, we did not claim that TORC1 is most active in GSCs. In fact, our data show that it is active in germ cells across the nanos-expressing domain, which we referred to collectively as “early germ cells”.

Both readouts showed similar activation pattern of TORC1 (as described line 281). Because the anti-pS6 and anti-p4E-BP antibodies are both produced in rabbit, it is not possible to perform a double immunostaining. To avoid any potential misinterpretation, we replaced the image in S1L Fig with a more representative image, in which p4E-BP is seen in germ cells at different stages of differentiation within the nanos-expressing domain where 4E-BP is overexpressed.

Of note, because S6 and 4E-BP are independent targets of mTor kinase, potential differences in their phosphorylation pattern could be due to reasons independent of TORC1 activity, such as variations in expression of the target protein itself or cell type dependent activity of phosphatases for example.

4. One of the major pieces of evidence to suggest differentiation of GSCs or GBs into transit amplifying spermatogonia is disrupted in mTor KD is the accumulation of spherical fusomes. However, this finding is presented as a single representative image that is left unclear if it demonstrates a typical or exceptional example. Quantification of the number of germ cells connected by each fusome structure would more clearly demonstrate the accumulation of cells that have failed to enter interconnected transit amplification in the mTor KD condition.

We now provide a quantification of the number of spherical fusomes per testis (Fig 2E), discussed in the text lines 314-317.

This shows a statistically significant (****) increase in germ cells with spherical fusomes upon downregulation of TORC1 (with *mTor^RNAi1^*, *mTor^RNAi2^* and *raptor^RNAi^*). This clearly demonstrates, together with the accumulation of pMad+ germ cells and additional markers discussed below, that differentiation of GSCs/GBs into transit amplifying spermatogonia is disrupted.

5. The increase in pMad+ cells upon TORC1 inhibition is clearly demonstrated and intriguing. The authors interpret this as ‘delayed differentiation’: if it is the case, STAT activation may still be limited to GSCs. Examining STAT upon TORC1 inhibition will be an important comparison to validate (or modulate) their claim to more accurately describe the impact of TORC1 inhibition. It may also be beneficial to include an additional output of cell stage to detect delayed differentiation, such as testing for an increased portion of nos+ germ cells or a decrease in Bam+ germ cells.

We thank the reviewer for these suggestions. We have stained control and mTor-depleted testes with an anti-phosphorylated STAT (pSTAT) antibody. While in control testes the staining was limited to the germ cells around the hub, mTor-depleted germ cells with pSTAT staining were detected several cell diameters away from the hub. Quantification indicated that depletion of mTor in adult early germ cells causes an increase in pSTAT-positive germ cells. These results are now presented in supplementary figure 2S-U and discussed in the revised manuscript lines 365-370.

We also stained for Bam. The protein was readily detected in all control testes (in 8 and 16-cell cyst) but was only rarely detected in mTor-depleted testes, as shown in the new supplementary figure 2S-U and discussed in the revised manuscript lines 361-364.

Taken together, our observations indicate that depletion of mTor causes an accumulation of GSC-like cells.

**Reviewer #2:**

In this manuscript, the authors demonstrate a previously unappreciated role for mTOR in differentiation of germ cells in the Drosophila testis. While mTOR was previously shown to be required in female germ cells and somatic cells of the testis, it was unknown whether it played a role in male germ cells. The work presented here fills this gap and shows that germ cell-specific mTOR loss results in blocked differentiation, as well as other phenotypes including accumulation of autolysosomes and mis-coordination between the soma and germline. Although mostly descriptive, this work does contribute valuable new knowledge and understanding of the pathways involved in germ cell differentiation.
The work presented here is sound and rigorous and results are backed up by the use of multiple RNAi lines for mTor and consistent results with knockdown of Raptor as well as Rapamcycin treatment, giving confidence in their findings. Finally, in addition to the main message of this work, that mTOR is involved in germline differentiation, several interesting points are raised that deserve publication, for instance that mTOR may have different targets or readouts in different tissues or that mTOR activity is involved in coupling differentiation between germline and soma. These findings will be important for the community to build on and provide mechanistic insights into germ cell differentiation in the testes.

Addressing the following points would improve the manuscript greatly and ensure that all the conclusions are adequately supported:

1. The authors claim that mTORC1 activity is regulated at least in part by the expression of mTor itself. This argument is based solely on the expression pattern of an mTor::GFP transgene, which does not fully rescue lethality associated with mTor mutation. This lack of full rescue could be due either to the tag preventing the protein from being fully functional, as discussed by the authors, or by the transgene failing to recapitulate endogenous expression fully. In the latter case, the lack of expression of the GFP construct may not reflect endogenous expression, and it is hard to draw very strong conclusions from these data. Could the authors provide further validation, for instance by in situ hybridization, or by examining read counts in the recent Fly Cell Atlas, to show that indeed mTor expression is low in spermatocytes? Failing that, the authors should tone down their conclusions (for example on l. 155: “TOR activity is regulated at least in part through differential expression of the mTor kinase” could be replaced with “TOR activity may be regulated…”), and could consider moving these data to the supplement, as they do not constitute the major part of their findings but instead distract from them.

Please see response to Reviewer 1- point #1.

In addition, as suggested, we replaced “TOR activity is regulated at least in part through differential expression of the mTor kinase” with “TOR activity may be regulated at least in part through differential expression of the mTor kinase” (line 285).

2. The loss of differentiated germ cells upon mTOR knockdown could be due to a differentiation arrest or alternatively to death of the differentiated cells. The latter possibility is not addressed in the experiments and cannot be formally excluded. While the lack of lysotracker-positive germ cell cysts would suggest that there is no increase in death of differentiated germ cells, it could be that the time point at which lysotracker was examined is too late to capture death that would occur soon after knockdown. This possibility could be acknowledged in the text.

We favor differentiation arrest due to the abnormally high number of germ cells with spherical fusomes, high pMad, and other markers that we have now assessed (accumulation of pSTAT+ germ cells, decrease in Bam expression, presence of single dividing germ cells away from the hub), all suggesting that GSCs do not differentiate properly. As pointed out by the reviewer, we cannot completely exclude the possibility that differentiated cells are dying, as we agree that the absence of lysotracker staining cannot rule out this possibility as cell death could occur rapidly after induction of the knock-down. In addition, we did not detect any cell death using cleaved Caspase 3 staining, as shown in the example below (n=10 for each condition).

3. A hallmark of GSCs and CySCs is their ability to proliferate – in the case of GSCs, the presence of proliferating cells that are not synchronized with their neighbors, and simply proliferation in the case of CySCs. It would be helpful to assess proliferation upon germline mTOR knockdown, to determine a) whether the ectopic GSC-like cells do indeed behave like GSCs, and b) whether the ectopic Eya expression in somatic cells does indicate a loss of CySCs.

Upon the reviewer’s recommendation, we have assessed proliferation using phospho-histone H3 staining. We now provide an example in supplementary Fig S2Q-R (discussed in the text lines 370-374), showing a single dividing germ cell several cell diameters away from the hub in a mTor-depleted testis. This indicates that the germ cells that accumulate upon mTor depletion indeed retain GSC/GB features (in addition to the fact that they have spherical fusomes, accumulation of pMAD and pSTAT).

We also performed EdU incorporation assay to label cells that underwent DNA replication in a 30 minutes incorporation pulse. We found that this method was better suited to label CySCs that were rarely visible with phospho-histone H3 staining. We did not observe any significant difference in the number of EdU positive CySCs (Vasa negative cells in contact with the hub) between control testes and testes expressing *mTor^RNAi^* in early germ cells. Similarly, expression of another marker enriched in CySCs, Patched (Ptc), was also found to be maintained in testes with germline specific mTor depletion (Supplementary Fig 4F). These results suggest that at least some CySCs are maintained, even though the differentiation marker Eya is prematurely expressed in cyst cells around the hub. We added these results in Supplementary Figure 4 G-I and they are now described in the text lines 540-541.

4. The authors show that in control GSCs, autophagosomes stain yellow with GFP-mCherry-Atg8a, suggesting that they do not acidify. Similarly, mTOR knockdowns lead to yellow autophagosomes, albeit larger. Thus, although the data clearly show an accumulation of large autophagosomes upon mTOR knockdown, whether they are dysfunctional is debatable, as the main difference with controls appears to be the size and number rather than function of the autophagosomes.

Indeed, as pointed out by the reviewer, the rare autophagosomes observed in control germ cells appear yellow, suggesting that they are not properly acidified and therefore not functional, whereas the autophagosomes observed in somatic cells, including hub cells, are mostly red and functional. This suggests that autophagy may not be active in germ cells under homeostatic conditions, which is in agreement with previous findings from our team (Sênos Demarco et al., 2020) showing that depletion of Atg proteins in the germline does not lead to any obvious germ cell phenotype. We now refer to these findings lines 471-472. Our new observation that autophagy is still not functional when TORC1 is inhibited suggests that TORC1 is not the main factor preventing autophagy in germ cells under homeostatic conditions. However, there is clearly an accumulation of abnormally large autophagolysosomes in that context, which could potentially contribute to other phenotypes caused by TORC1 inhibition, for example by sequestering factors required for germ cell differentiation.

5. As a minor point, some of the data shown are discontinuous variables (such as number of cells, for example Fig. 2G, 4C,4F) and are therefore by definition not normal. For these, a non-parametric test should be conducted (Mann-Whitney), to avoid making assumptions on data normality. In practice, this will most likely not change the outcomes of whether the changes observed are statistically different.

Thank you for raising this point. We conducted Mann-Whitney tests instead of Student t-tests when appropriate (Main figures 2E, 2H, 4C and 4F and Supplementary figures S1J, S2C, S2U, S2V and S4I). We corrected the figure legends and the Material and Methods section accordingly.
