# Supplementary material for: mTORC1 is required for differentiation of germline stem cells in the Drosophila melanogaster testis
Source: PLoS One. 2024 Mar 21;19(3):e0300337. doi: 10.1371/journal.pone.0300337 (PMC10956854; doi:10.1371/journal.pone.0300337)
Supplement: Attachment — Submitted filename: Response to Reviewers.0124mc.docx [file pone.0300337.s007.docx]

We thank the Reviewers for their time and thoughtful comments that have helped improve our manuscript. Please see our responses below; changes to the manuscript are highlighted in yellow in the revised manuscript.

**Reviewer #1:**

The authors addressed most of comments to the previous round of review. However, their response regarding the use of mTor-GFP as expression reporter is not satisfactory. Subheading of the first result section says ‘Differential expression of mTor correlates with variations in TOR activity throughout germ cell differentiation’ using mTOR-GFP. In the response to the review, the authors claim that mTor-GFP expression pattern is not intended to serve as the foundational premise of this manuscript. It does not matter what data serve as a premise of study, but it is simply a ground rule of science that a claim must be supported by the results. If mTor-GFP transgene does not rescue mTor mutant, how would one know whether the reporter GFP pattern represents endogenous mTor expression pattern? We feel uncomfortable that the argument about what is the premise of the work interferes with more fundamental rule of evidence-based claim. At least the authors should be more transparent about mTor-GFP not functional in the main text, instead of the method section.

We apologize, as we didn’t understand the primary concern of the reviewer. We have now made several changes to the text and figures to highlight that the mTor-GFP reporter used in our study (also used in Gui et al, Cell Reports, 2023) is expressed in germ cells and does not appear to be expressed in somatic cells- at least not in the tissues we’ve surveyed. Accordingly, the reporter does not reflect the full scope of mTor expression or mTORC1 activity, which is consistent with the inability of the transgene to rescue the larval lethality associated with mTor loss of function alleles (Sarov et al., 2016). We have moved the note to this reference, originally in the Materials and Methods, to the main text (page 15, lines 267-270). We changed the subheading of the first section to “The TOR pathway is active in male GSCs and early germ cells,” and we revised the order of the data presented in Figure 1 to put more emphasis on where TOR is active in germ cells. Finally, we have included reference to Gui et al., a recently published study showing expression of the same *mTor::GFP* transgene in female early germ cells, which corroborates our findings in males. As we did not detect expression of *mTor::GFP* in somatic tissues in which TOR is known to be active and required, but we do see loss of GFP in response to expression of a *TorRNAi* transgene in germ cells (Supplemental Figure 1 K-L), we hypothesize that the TOR transgene may lack sequences that are necessary for expression in somatic tissues.

**Reviewer #2:**

Thank you for addressing all the comments raised previously. This manuscript will be helpful to the field as a whole. One minor typo you may want to correct in proofing: in the methods, "Rabbit monoclonal anti-phosho-SMAD1/5" should be "phospho".

Thank you for noting this typo. It has been corrected in the revised version.
